# Effect of Long-Term Music Training on Emotion Perception From Drumming Improvisation

**DOI:** 10.3389/fpsyg.2018.02168

**Published:** 2018-11-09

**Authors:** Martina Di Mauro, Enrico Toffalini, Massimo Grassi, Karin Petrini

**Affiliations:** ^1^Department of General Psychology, University of Padua, Padua, Italy; ^2^Department of Psychology, University of Bath, Bath, United Kingdom

**Keywords:** music training, expressiveness, emotion perception, valence, drumming

## Abstract

Long-term music training has been shown to affect different cognitive and perceptual abilities. However, it is less well known whether it can also affect the perception of emotion from music, especially purely rhythmic music. Hence, we asked a group of 16 non-musicians, 16 musicians with no drumming experience, and 16 drummers to judge the level of expressiveness, the valence (positive and negative), and the category of emotion perceived from 96 drumming improvisation clips (audio-only, video-only, and audiovideo) that varied in several music features (e.g., musical genre, tempo, complexity, drummer’s expressiveness, and drummer’s style). Our results show that the level and type of music training influence the perceived expressiveness, valence, and emotion from solo drumming improvisation. Overall, non-musicians, non-drummer musicians, and drummers were affected differently by changes in some characteristics of the music performance, for example musicians (with and without drumming experience) gave a greater weight to the visual performance than non-musicians when giving their emotional judgments. These findings suggest that besides influencing several cognitive and perceptual abilities, music training also affects how we perceive emotion from music.

## Introduction

Music can be found in different forms in all human cultures, and it is recognised as an important means of emotion communication. Research has established that musicians can successfully communicate emotions to an audience (Gabrielsson and Lindström, 1995; Gabrielsson and Juslin, 1996; Balkwill and Thompson, 1999; Juslin and Madison, 1999), however, the emotions perceived and/or felt by the listener depend on various individual factors (e.g., personality; Juslin et al., 2008).

Despite the clear effect of music practise on the listener/observer cognitive processes (e.g., Lee and Noppeney, 2011; Petrini et al., 2011) and on the ability to recognise emotions from speech prosody (e.g., Thompson et al., 2004; Strait et al., 2009; Lima and Castro, 2011b; Pinheiro et al., 2015), it is still unclear how musical practise affects perceived emotions and expressiveness from music. The present study contributes to the emerging literature regarding the relation between musical expertise and expressiveness/emotion perception from music (e.g., Bhatara et al., 2011; Lima and Castro, 2011a; Castro and Lima, 2014). This knowledge will increase understanding of the effects of music practise on emotional, cognitive, and perceptual processes and assess whether music can be used as an efficient and cost effective treatment for individuals with socio-emotional disorders (e.g., autistic and schizophrenic individuals).

To this end we examined whether individual differences in musical ability influence how emotions from music are perceived.

### Expression, Perception, and Induction of Emotions Through Music

Musicians are able to communicate specific emotions via their expressive performances while listeners generally perceive the intended emotions (Laukka and Gabrielsson, 2000). Despite the consistent level of agreement found among listeners when categorising the expressed or perceived emotion (Campbell, 1942; Juslin, 1997a), listeners’ agreement seems greater for some emotions (e.g., happiness, sadness) than others (e.g., jealousy; Juslin and Laukka, 2004). The debate about which type of emotions are induced and/or perceived through music is still ongoing. Some researchers argue that music can induce only broad positive and negative states (Clark, 1983), whereas others argue that music can induce a range of both basic and complex emotions (Gabrielsson, 2001; Juslin and Västfjäll, 2008). Some researchers argue that music can induce basic emotions (e.g., happiness, sadness, anger, fear, disgust, and surprise; Krumhansl, 1997), whereas others affirm that music can communicate only a limited set of states specific to music (e.g., amazement and peacefulness) but not common everyday emotions (e.g., shame and jealousy; Scherer, 2003; Zentner et al., 2008). A meta-analysis of 41 studies has shown that professional musicians are able to communicate efficiently five basic emotions (happiness, anger, sadness, fear, tenderness) to listeners (Juslin and Laukka, 2003). In contrast, they are not able to communicate complex emotions such as contentment, curiosity, and jealousy (Juslin and Lindström, 2003).

The ability to communicate emotions through music mainly depends on its similarity to other forms of non-verbal communication and the type of emotions that can be expressed through those channels (Clynes, 1977; Juslin, 1997b); for instance, basic emotions that can be accurately perceived through music seem to mirror those that can be perceived through speech (Juslin and Laukka, 2003), as music and speech use similar neurological mechanisms to convey emotions (Escoffier et al., 2013; Peretz et al., 2015; Paquette et al., 2018). However, some negative emotions (e.g., guilt, shame, jealousy, disgust, contempt, embarrassment, anger, and fear) are not commonly experienced through music (Zentner et al., 2008), because even sad or nostalgic music usually induces positive emotions (Juslin and Laukka, 2003; Laukka, 2007). Better yet, sadness as communicated through music can be usually perceived, i.e., sad music is perceived as sad, but does not usually induce sadness in the listeners (Zentner et al., 2000), in contrast, usually listening to sad music often evokes positive emotions (e.g., listening to a sad romantic song; Juslin and Sloboda, 2010).

Consequently, we asked whether music practise would affect the perception of basic emotions as well as the perceived valence and level of expressivity to reflect the complexity of the emotion communicated by the musician. Also because our aim was to examine whether music practise can affect how we perceive emotions communicated by others through music, we examined whether music practise affects the perception, rather than the feeling, of emotions from music performance.

### The Role of Individual Factors

To reach a good understanding of how listeners perceive emotions from music, it is also necessary to investigate contingent (Sloboda and Juslin, 2001) and individual factors (Abeles and Chung, 1996). Nevertheless, only a few studies have focused on the role of individual differences in the perception of emotions from music. For instance, being amusic or using cochlear implants affects the emotion judgements from music (Ambert-Dahan et al., 2015; Gosselin et al., 2015). Moreover, Lima and Castro (2011a) and Castro and Lima (2014) found that the number of the years of musical training is associated with more accurate recognition of the musical emotion. Yet little is known about whether the individual differences in musical training influence the perception of emotions from music and if so how. It is recognised that musical training and expertise affect the multisensory perception of music (e.g., Petrini et al., 2009a,b, 2010a, 2011; Lee and Noppeney, 2014). For example, trained musicians (e.g., drummers and pianists) and non-musicians differ in their sensitivity to desynchronisation between the movement of a musician and the resulting sound (e.g., Lee and Noppeney, 2011; Petrini et al., 2011). However, despite the consistent findings showing a strong effect of long-term music and dance training on several cognitive and perceptual abilities (e.g., Scherer, 2003; Petrini et al., 2009a,b, 2010a; Lee and Noppeney, 2014) and their subtending neural mechanisms (e.g., Calvo-Merino et al., 2005; Petrini et al., 2011; Lee and Noppeney, 2014; Lu et al., 2014), it is still unclear whether the effect of musical training extends to the perception of emotions from music.

Here we investigate how different types of musical training and the absence of such training affect how emotions from musical performance are perceived.

### How Musicians Communicate Emotions From Music

Sound plays a dominant role in the communication of emotions through music (Vines et al., 2006). The various expressive intentions of musicians implicate a change in the acoustic cues (e.g., dynamic, timing, tempo, mode, pitch, harmony, loudness; Laukka and Gabrielsson, 2000; Juslin, 2001; Gabrielsson and Juslin, 2003; Gagnon and Peretz, 2003; Juslin and Laukka, 2003; Juslin and Lindström, 2003). For instance, performers communicate happiness by using major mode and sadness by using minor mode (Hevner, 1935; Gerardi and Gerken, 1995; Peretz et al., 1998; Gagnon and Peretz, 2003); note that, in the theory of Western music, mode refers to a sequence of tones and semitones arranged according to a specific order. Among all acoustic features, tempo seems to be one of the most important variables used to determine emotions and expression in music (Hevner, 1937; Rigg, 1964; Gagnon and Peretz, 2003) from early childhood (Dalla Bella et al., 2001). For example, slow melodies communicate sadness whereas fast melodies communicate happiness, fear, and anger (Juslin, 2001; Gabrielsson and Juslin, 2003; Gagnon and Peretz, 2003; Juslin and Laukka, 2003; Juslin and Lindström, 2003; Juslin, 2009). Moreover, musicians might communicate their emotion intentions also through musical genre, as, for instance, heavy metal music helps its fans to regulate sadness, to reduce anger and to enhance positive emotions (Sharman and Dingle, 2015).

In addition to sound, when a musical performance is also transmitted visually (such as when listeners watch a live music performance), music becomes a richer emotional experience. Besides the acoustic aspects of the music performance, facial expressions, and gestures of musicians influence the emotions perceived by listeners (Davidson, 1993; Thompson et al., 2008). For example, it has been shown that marimbists, saxophonists, and bassoonists were able to communicate specific emotions through body movements alone (Dahl and Friberg, 2007). More specifically, musicians may communicate different emotions depending on the movements they use when playing a musical instrument. For instance, the authors (Dahl and Friberg, 2007) showed that large and fast movements communicated happiness whereas small and slow movements communicated negative emotions.

Similarly, it has been shown that when the sound and video of a clarinettist performance were presented together to a group of musicians, the visual information supported, modified, or confirmed the emotional content perceived through the sound (Vines et al., 2006). However, in a different study, this effect of visual information over sound did not extend to non-musicians, who relied more on the sound when perceiving the emotional content from a drummer’s and saxophonist’s performance (Petrini et al., 2010b). These separate studies suggest that the weight given by musicians to the emotional visual information may be greater than that given by non-musicians. To examine this possibility, here we investigate how musicians and non-musicians perceive emotion from music when solely listening, solely observing, or both listening and observing such performances. Also because it is still unclear whether long-term musical training affects how sound and body features are used to perceive emotions we tested how changes in tempo, musical genre, musician’s expressiveness, musician’s style, and level of complexity of musical pieces affected the perceived emotions.

### Melodic vs. Rhythmic Music

It is understood that melody modulates the perception of emotions from music (e.g., Gagnon and Peretz, 2003). Conversely it is less well understood how, rhythm, the other component of music impacts emotional perception despite findings that have shown that some emotions expressed through drums can be recognised with high accuracy by non-musicians (Petrini et al., 2010b). The drums and percussions allow more evident and less restricted upper body movements than those permitted by other instruments (Petrini et al., 2010b) and are easily learned and reproduced from an early age with tangible consequences on perceptual and cognitive abilities (Gerson et al., 2015). For example, it was shown that preverbal infants engage in rhythmic behaviour more often for music rather than speech and this engagement to music is associated to the level of positive behaviour (smiles) by the infant (Zentner and Eerola, 2010). Similarly, it was shown that practising with the drums for only 5 min increases six-month-old infants’ ability to detect auditory and visual desynchronisation (Gerson et al., 2015). Hence, playing a rhythmic instrument could be an effective means of therapy from an early age if pure rhythmic instruments can communicate emotional states. For these reasons, we chose to focus on drumming because it is a purely rhythmic instrument and the associated rhythmic and timing skills have been shown to have positive effects on the language skills of children with dyslexia (Overy, 2003). Similarly, that interventions using the drums have been shown to facilitate speech in non-verbal children with autism (Wan et al., 2011).

### The Present Study

The main aim of the present study was to examine whether musical training affects the way emotions from drumming are perceived. As it is an exploratory study, specific hypotheses were not formulated, however, we asked whether: (1) musicians would perceive emotion from rhythmic music differently from non-musicians; (2) whether the level of familiarity with the instrument used in the music performance would affect the way emotions are perceived; (3) whether musicians would be affected more than non-musicians by changes in certain characteristics of the music performance, such as musical genre, tempo, complexity, drummer’s expressiveness, drummer’s style, and sensory modality (i.e., whether musicians would weigh the visual information from the music performance more than non-musicians).

To this end, we asked participants with different levels and types of musical expertise (drummers, other musicians with no drumming experience, and non-musicians) and familiarity with the musical stimulus (as the performance was drumming improvisations) to rate the level of expressiveness of music clips under different sensory conditions (audio-only, video-only, audio with video), judge the positivity (or negativity) of the perceived emotion, and categorise the perceived emotion among a group of basic emotions.

## Materials and Methods

### Participants

The study involved 48 voluntary adults recruited through social media. The number of participants is similar or higher than previous studies investigating the effect of long-term music training on cognitive and perceptual abilities (e.g., Petrini et al., 2010a, 2011; Bhatara et al., 2011; Lee and Noppeney, 2014; Lu et al., 2014). These studies generally reported medium to very large effect sizes on behavioural data (e.g., accuracy or response times in the detection of audiovisual asynchronies). Although in most cases they are not reported explicitly, they can be derived from figures or descriptive data, and seem to indicate differences between musicians and non-musicians, or equivalent effects of music training on sensitivity to emotions in music, of about Cohen’s d ≈ 0.50–1.50 (e.g., Bhatara et al., 2011; Lima and Castro, 2011a; Castro and Lima, 2014; Lee and Noppeney, 2014; Lu et al., 2014) or about 1.50–2.00 (Petrini et al., 2010a; see their Figure 5, and refer to the binomial distribution). Expecting a Cohen’s d of 1.00 in a between-group comparison, power of 0.80, and a significance level of 0.05, the estimated *N* should be of 16–17 (as calculated, for example, using the R software’s pwr library).

Sixteen participants were non-musicians – ranging from 21 to 43 years of age and seven females (*M* = 29.37; *SD* = 6.66); sixteen were musicians with no drumming experience – ranging from 21 to 43 years of age and eight females (*M* = 27.81; *SD* = 5.20); sixteen were drummers – ranging from 20 to 44 years of age and eight females (*M* = 29.5; *SD* = 7.72). The non-musicians had never played a musical instrument except for the basic music classes of the Italian Middle School curriculum. Non-drummer musicians had played a musical instrument, except drums, for at least 4 years (years of music training: *M* = 14.06; *SD* = 7.14). Drummers had been playing only drums for at least 4 years (years of music training: *M* = 15.81; *SD* = 9.33).

All participants reported normal hearing, and normal (or corrected to normal) vision. All participants gave their written informed consent before testing began. The study received ethical approval from the Ethics Committee of the Department of Psychology, University of Padua (n° 2425).

### Stimuli and Apparatus

**FIGURE 1:**
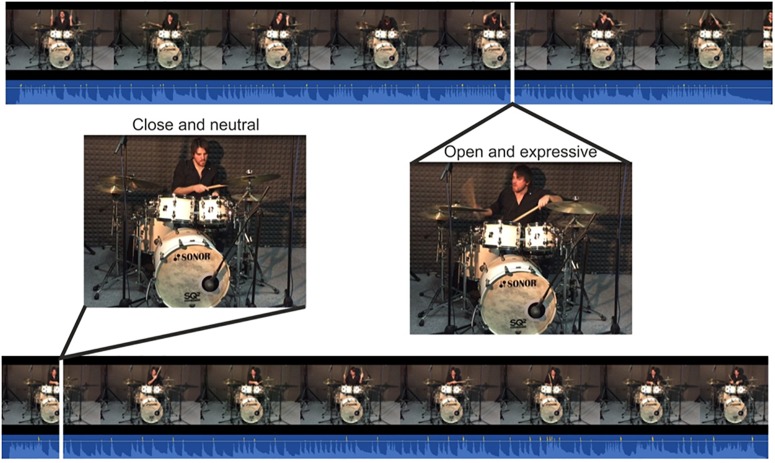
Example of clips used in the present study. On the left, frame and waveform sample from closed arms and neutral performance; on the right, frame and waveform sample from open arms and expressive performance.

The stimuli were audio-visual clips of the performances of a professional drummer (years of music training: 25; years of teaching: 16 – see Figure 1). We asked the drummer to improvise each recorded performance. Improvisations were chosen instead of known musical pieces to avoid any effect of familiarity and episodic memory on participants’ perceived emotions (Juslin and Västfjäll, 2008; Petrini et al., 2010b). In addition, no instructions concerning specific emotional intentions were given to the drummer, whereas the features of the performances were manipulated. This was done to compare our results with those of studies using the drums (Petrini et al., 2010b) and asking the drummers to specifically communicate some basic emotions (which reduces the complexity and the realism of the music performance). Hence, performances were different for musical genre (jazz or heavy metal), complexity (complex or simple rhythms), tempo (60 or 120 beats per minute), drummer’s expressiveness (with minimal or maximum expressive interpretation of the music), and drummer’s style (playing with open or crossed arms). Matching the levels of these factors, 32 different stimuli were obtained. Initially, we asked drummer to repeat each performance five times to have an initial measure of the drummer’s level of consistency among the repeated performances. Since the drummer was a technically accurate professional, the different clips of the same stimulus resulted in a consistent visual output (e.g., similar grooves, timing, and dynamics). However, we selected only one clip among five. First, we eliminated any clips with minor technical errors (e.g., video camera not perfectly facing the performer) and then we choose the most accurate clip in terms of drumming technique (minor details, e.g., drumsticks grip, fills). Therefore, despite recording a total number of 160 audio-visual performances, only 32 of these were selected for the experiment. Then, each audio-visual recording was also converted into an audio-only stimulus and video-only stimulus, thus reaching a total number of 96 clips. Each clip had a duration of about 40 s with the audio-only stimuli replacing the video of the drummer with a black screen and the visual-only stimuli muting the audio track. We chose not to use different types of clips for the audio-only, video-only, and audiovisual conditions to not risk a possible confound, as the various clips could differ in expressing a certain emotion. This ensured that any significant effects of the modality could be tied to differences in sensory information rather than differences in quality of the music performance.

The original audio-visual clips (see example of Videos in Supplementary Material) were recorded in a professional music studio using Sonor SQ2 drums and Ufip cymbals. The video recordings were made with an iPhone 6, facing the performer. For the audio recording we used three microphones XXL (two overhead and one for drum bass), mixer/sound card (M-Audio Fast Track Ultra 8R), and Pro Tools 11 programme. The video-track and the audio-track were combined together by using Adobe Premier Pro 2.0. The audio-visual files (Mpeg 1920 × 1080) were converted into avi 1366 × 768 files using Freemake Video Converter. The stimuli were presented to participants via E-Prime 2.0 (Psychology Software Tools, Inc.) on a Sony Vaio laptop computer. In audio-visual and audio-only stimuli, the audio was delivered through Sennheiser HD 280 Pro (64 ohms) headphones.

### Procedure

Participants were tested individually in different rooms with testing always conducted in a quiet room of similar size. All participants were informed about the procedure before testing. Successively, the 96 clips were presented to participants, who were then asked, after each clip, (i) to rate the level of perceived expressiveness by using a 7-points Likert scale (1: little expressive – 7: very expressive), (ii) to judge on the positivity (or negativity) of the perceived emotion with a dichotomous response, and (iii) to choose the perceived emotion by selecting one from seven possible emotion categories: six basic emotion (happy, sadness, anger, fear, disgust, and surprise) plus a neutral emotion for participants who could not readily categorise the perceived emotions among the basic emotions or if they did not perceive emotion. To answer these questions, participants had to use the number pad of the laptop. Questions and stimuli were presented in random order. This randomised method, that has been used in similar musical and non-musical studies (e.g., Collignon et al., 2008; Piwek et al., 2015) to assess the benefit of multisensory perception for emotion processing, was used in order to reduce the effect of learning and fatigue, and thus reduce the possibility that any benefit found for the audio-visual clips was due to increased familiarity with the audio-only and visual-only clips. Moreover, participants could take a break after every 32 stimuli. Upon completion of the testing procedure, we gathered information relating to each participant via a questionnaire. For the purpose of the present research, we used the answers to three questions, i.e., whether they played any instruments, if they did play an instrument which instruments they played and for how long they practise with these instruments.

## Results

Because the response variables were repeated measurements (by participant and by clip) for the ratings of perceived expressiveness, perceived positivity, and perceived emotion category data were analysed using mixed-effects linear models. In particular, because the perceived positivity and the perceived emotion category consisted of dichotomous data (1: “yes/perceived,” 0: “no/not-perceived”), these variables were analysed using logistic regression models (i.e., with a logit link function). The package “lme4” (Bates et al., 2015) of the R software was used to compute the models. The graphics were obtained using the package “effects” (Fox, 2003).

Our main aim was to examine how musical practise contributed to the perception of emotion from music. The fixed effects entered in the models were: group as a between-subject factor (3 levels: non-musicians, non-drummer musicians, or drummers), and musical genre (2 levels: jazz or heavy metal), tempo (2 levels: 60 or 120 beats per minute), drummer’s expressiveness (2 levels: with minimal or maximum expressive interpretation of the music), sensory modality (3 levels: audio-only, video-only, or audio-visual clips), complexity (2 levels: complex or simple rhythms), and drummer’s style (2 levels: open or crossed arms) as within-subjects factors. Participants and clips were treated as random effects, with random intercepts, in all models.

The main effect of each group and all the two-way interactions between each group and the other factors were examined for each response variable (to examine the higher–order interactions or other main effects, the data can be found online, doi: [doi:10.6084/m9.figshare.7262180.v1]). The significance of each effect was assessed using a likelihood ratio test for nested models based on the chi-square distribution (Pinheiro and Bates, 2000). This test compares the likelihood of two models that are identical except for the fact that one excludes and the other includes a given effect. The interactions were tested by adding them, one at a time, to the model fully inclusive of all main effects. A summary of the main effect of each group and its two-way interactions with all the other factors are reported for each response variable, along with models’ parameters, in Table 1. These effects are described below, separately by response variable. The interpretation of the significant effects was based on visual inspection of the figures (reporting estimated mean values or probabilities and 95% confidence intervals), and the consideration of model parameters (Table 1) in case of ambiguity.

**Table 1:** Summary of mixed-effects models on perceived expressiveness, emotional valence, and emotion categories perceived above chance.

Fixed effect	Perceived expressiveness				Emotional valence				Emotion:“Neutral”				Emotion:“Happiness”			
	χ^2^(df)	*B*	*SE*	*p*	χ^2^(df)	*B*	*SE*	*p*	χ^2^(df)	*B*	*SE*	*p*	χ^2^(df)	*B*	*SE*	*p*
Group	8.53 (2)			0.014	1.54 (2)			0.462	4.32 (2)			0.116	6.85 (2)			0.033
Drummers		0.17	0.32	0.591		0.38	0.38	0.311		0.37	0.29	0.200		0.27	0.24	0.258
Non-drummer musicians		0.89	0.32	0.007		0.43	0.38	0.255		-0.24	0.29	0.410		-0.38	0.24	0.114
Group × Music genre	81.75 (2)			<0.001	41.31 (2)			<0.001	6.79 (2)			0.034	11.38 (2)			0.003
Heavy metal:Drummers		0.33	0.10	<0.001		0.78	0.19	<0.001		-0.42	0.16	0.010		-0.59	0.20	0.004
Heavy metal:Non-drummer musicians		-0.53	0.10	<0.001		-0.45	0.18	0.013		-0.16	0.17	0.343		-0.66	0.22	0.003
Group × Tempo	13.61 (2)			0.001	17.63 (2)			<0.001	1.08 (2)			0.582	4.48 (2)			0.107
120 beats:Drummers		0.22	0.10	0.027		0.70	0.18	<0.001		-0.07	0.16	0.684		0.17	0.19	0.389
120 beats:Non-drummer musicians		-0.13	0.10	0.173		0.05	0.18	0.775		-0.17	0.17	0.298		0.45	0.21	0.035
Group × Expressiveness	3.05 (2)			0.218	1.79 (2)			0.408	0.077 (2)			0.963	0.88 (2)			0.643
Expressive:Drummers		-0.17	0.10	0.089		0.11	0.18	0.535		0.01	0.16	0.934		-0.15	0.19	0.436
Expressive:Non-drummer musicians		-0.11	0.14	0.419		0.28	0.21	0.179		-0.05	0.24	0.826		-0.19	0.23	0.409
Group × Modality	15.74 (4)			0.003	13.44 (4)			0.009	7.51 (4)			0.111	17.63 (4)			0.001
Audio only:Drummers		-0.08	0.12	0.520		0.14	0.23	0.540		0.06	0.20	0.748		-0.01	0.22	0.949
Audio only:Non-drummer musicians		0.02	0.17	0.908		0.28	0.27	0.293		0.14	0.29	0.632		0.03	0.26	0.910
Video only:Drummers		0.30	0.12	0.013		0.71	0.22	0.001		0.36	0.20	0.066		0.85	0.25	<0.001
Video only:Non-drummer musicians		0.36	0.13	0.008		0.58	0.23	0.011		0.52	0.23	0.023		0.83	0.27	0.003
Group × Complexity	6.59 (2)			0.037	0.46 (2)			0.795	5.09 (2)			0.078	0.14 (2)			0.933
Complex:Drummers		0.24	0.10	0.014		-0.10	0.18	0.587		-0.36	0.16	0.024		0.06	0.19	0.768
Complex:Non-drummer musicians		0.08	0.14	0.572		0.03	0.21	0.880		-0.22	0.24	0.353		-0.02	0.23	0.941
Group × Drummer style	1.69 (2)			0.429	1.13 (2)			0.568	2.47 (2)			0.291	7.24 (2)			0.027
Crossed arms:Drummers		0.13	0.10	0.199		0.13	0.18	0.457		-0.20	0.16	0.216		0.49	0.19	0.010
Crossed arms: Non-drummer musicians		0.05	0.14	0.706		-0.08	0.21	0.692		0.12	0.24	0.622		0.10	0.23	0.658

*For emotional valence and emotion categories (neutral and happiness) a logistic regression analysis was used. Baseline categories were: “non-musicians” for Group, “jazz” for Music genre, “60 beats” for Tempo, “neutral” for Expressiveness, “audiovisual” for Modality, “simple” for Complexity, “open arms” for Drummer style.*

### Perceived Expressiveness

A preliminary analysis was conducted to ascertain a high level of consistency among participants’ responses for this variable on the 7-points Likert scale. Cronbach α = 0.88 indicated good consistency among participants in reporting perceived expressiveness.

**FIGURE 2:**
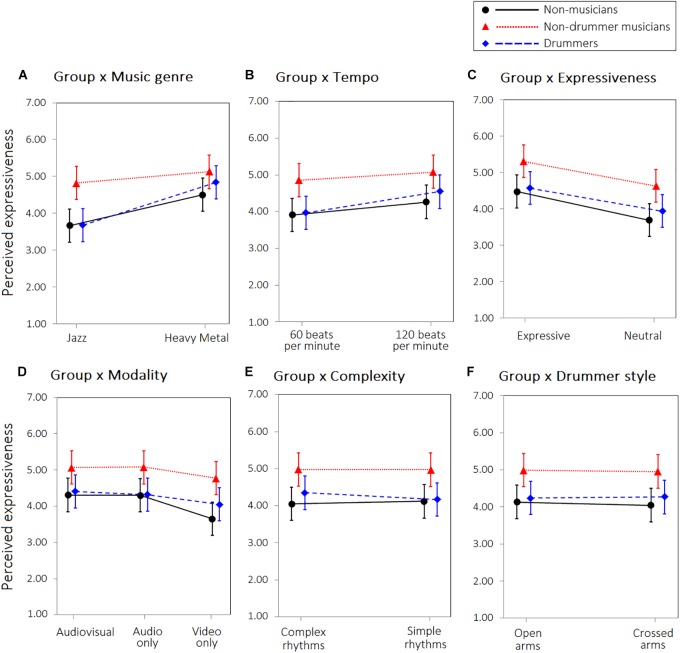
Interaction between Group and **(A)** Music genre, **(B)** Tempo, **(C)** Expressiveness, **(D)** Modality, **(E)** Level of difficulty, **(F)** Drummer style, on the perceived expressiveness of the clips. Error bars represent 95% confidence intervals of the estimated means.

A significant main effect of group was found. As can be seen in Figure 2, non-drummer musicians gave higher expressive judgments than non-musicians or drummers. A significant interaction between group and musical genre was also found, with non-musicians and drummers showing a higher level of perceived expressiveness in heavy metal than jazz clips, whereas non-drummer musicians showed a high or similar level of expressiveness in both types of clips (Figure 2, panel A). A significant interaction was found also between group and tempo: the perceived expressiveness was higher for “120 beats per minute” than for “60 beats per minute” performances, but this effect was stronger in drummers (Figure 2, panel B). A significant interaction between group and modality was also found: the three groups perceived the video-only modality as less expressive than the audio-only and audio-video conditions, but this difference was more prominent in non-musicians than in drummers or non-drummers’ musicians (Figure 2, panel D). Finally, a significant interaction between group and complexity was found, with complex performances perceived as more expressive than simple performances only by drummers. No other significant interactions were found. Model parameters are reported in Table 1.

### Emotional Valence

**FIGURE 3:**
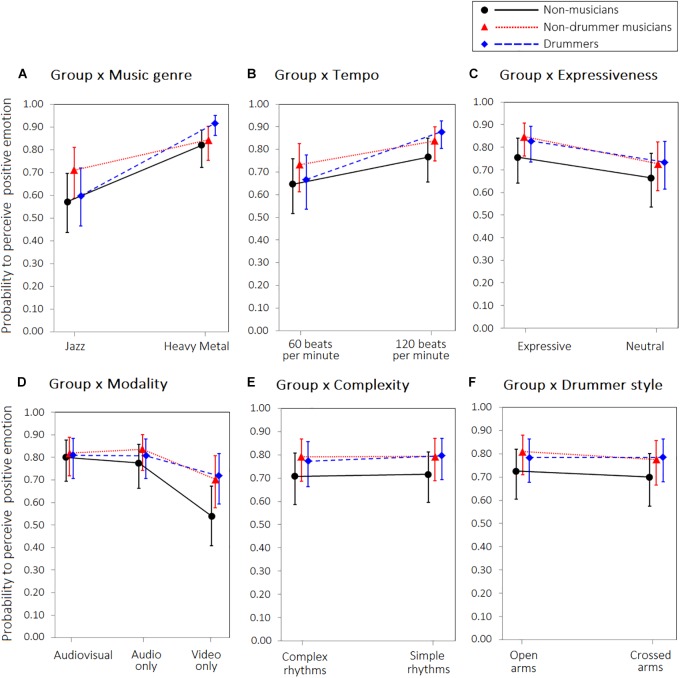
Interaction between Group and **(A)** Music genre, **(B)** Tempo, **(C)** Expressiveness, **(D)** Modality, **(E)** Level of difficulty, **(F)** Drummer style, on the probability to perceive a more positive (as opposed to more negative) emotion. Error bars represent 95% confidence intervals of the estimated probabilities.

As all responses were of a binomial type (1: “positive”; 0: “negative”), logistic regressions (i.e., generalised linear models with a logit link function) were computed. We did not find a significant main effect of group. However, a significant interaction between group and musical genre was observed, with all three groups perceiving a more positive emotion in heavy metal than jazz clips, although this difference was less prominent in the non-drummer musicians (Figure 3, panel A). A significant interaction between group and tempo was also found: although a more positive emotion was reported for performances at “120 beats per minute” than those at “60 beats per minute,” this difference appeared stronger in drummers than in the other participants (Figure 3, panel B). Finally, a significant interaction between group and modality was found: although the video-only modality was perceived less positively than the audio-only and audio-video conditions by all groups, this was especially evident for non-musicians (Figure 3, panel D). No other significant interactions were found. Model parameters are reported in Table 1.

### Emotion Category

We asked participants to choose the perceived emotion by selecting one of seven emotion categories (neutral, happy, sadness, anger, fear, disgust, and surprise). The most chosen emotion was neutral (37.48%), followed by happiness (20.40%), sadness (12.46%), surprise (10.63%), anger (9.51%), disgust (5.51%), and fear (4.01%). Since we had seven emotions in total, each had 14.28% (100/7 = 14.28) chance to be chosen randomly. As a result, we only discussed the ones that exceed the level expected from a uniform distribution of responses, such as neutral (37.48%) and happiness (20.40%). We analysed and discussed each emotion separately, considering each of them as binomial data (if that emotion was chosen: 1, or if that emotion was not chosen: 0). Therefore, to analyse the data we again used logistic regressions.

**FIGURE 4:**
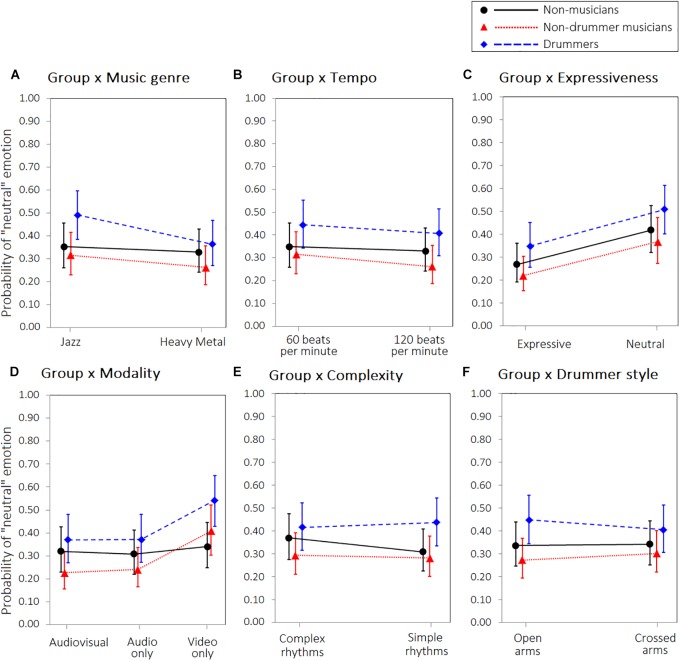
Interaction between Group and **(A)** Music genre, **(B)** Tempo, **(C)** Expressiveness, **(D)** Modality, **(E)** Level of difficulty, **(F)** Drummer style, on the probability to experience “neutral” emotion. Error bars represent 95% confidence intervals of the estimated probabilities.

For the neutral emotion we did not find a significant main effect of the group. However, we observed a significant interaction between group and musical genre: heavy metal was less likely to convey neutral emotion than jazz, but this effect was stronger in drummers (Figure 4, panel A). No other interactions reached statistical significance. Model parameters are reported in Table 1.

**FIGURE 5:**
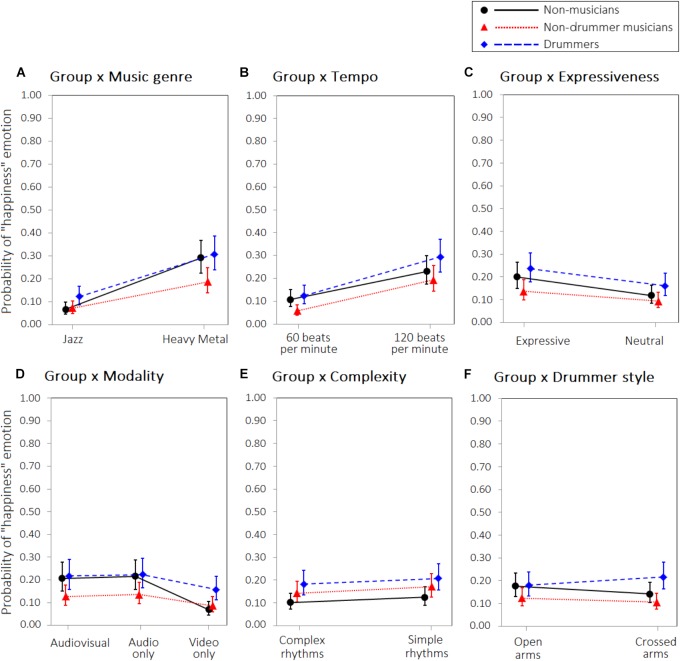
Interaction between Group and **(A)** Music genre, **(B)** Tempo, **(C)** Expressiveness, **(D)** Modality, **(E)** Level of difficulty, **(F)** Drummer style, on the probability to experience “happiness” emotion. Error bars represent 95% confidence intervals of the estimated probabilities.

For happiness we observed a significant main effect of the group. As can be observed from the parameters in Table 1, drummers had a higher probability of perceiving happy emotions than non-musicians, whereas non-drummer musicians perceived less happiness than non-musicians. However, none of the two parameters were significant, suggesting that the significant difference between groups can only be explained by the greater level of happiness perceived by drummers when compared to non-drummer musicians. A significant interaction between group and musical genre was observed: although happiness was more likely to be perceived for heavy metal than jazz, this difference was more prominent for non-musicians than for the other groups (Figure 5, panel A; parameters in Table 1). A significant interaction was also found between group and modality: although the video-only modality was less likely to convey happiness than the audio-only and audio-video conditions, this effect appeared stronger in non-musicians than in the other groups (Figure 5, panel D). Finally, a significant interaction between group and drummer’s style was observed with crossed arms more likely to convey happiness in drummers but not in the other two groups (Figure 5, panel F). No other significant interactions were found. Model parameters are reported in Table 1.

An additional analysis was conducted on the association between years of music training and emotional perception from music. Given the limited size of the groups, we pooled drummers and non-drummer musicians together. Note that the between-group difference in terms of years of music training was negligible, Δ*M* = 1.75 years, *t*(30) = 0.59, *p* = 0.56. The analysis was conducted using the same (generalised) mixed-effects linear models described above, but adding years of music training as another predictor. Due to the exploratory nature of this analysis, we set a critical α = 0.005 for significance. Effect size was reported as the odds ratio for a 5-year increase in music training (OR^5y^) or the model parameter *B* (again for a 5-year increase in music training) depending on the response variable.

Out of the four response variables (perceived expressiveness, emotional valence, “neutral” emotion, and “happiness” emotion), years of music training was negatively associated with the choice of happy emotion, χ^2^(1) = 9.00, *p* = 0.003, OR^5y^ = 0.80; this main effect was explained by an interaction with musical genre, χ^2^(1) = 10.88, *p* < 0.001, such that the negative association between years of music training and the choice of happiness as category was greater for jazz than for metal clips, OR^5y^ = 0.78. For perceived expressiveness, we found a significant interaction between years of music training and musical genre, χ^2^(1) = 56.90, *p* < 0.001 (i.e., years of music training was negatively associated with perceived expressiveness for jazz but not for metal, B^5y^ = -0.21), and a significant interaction between years of music training and modality, χ^2^(1) = 21.35, *p* < 0.001 (i.e., years of music training was negatively associated with perceived expressiveness for the video-only modality, B^5y^ = -0.18, but not for the other two modalities). No other significant main effects or interactions were found.

## Discussion

In the current study, we asked non-musicians, drummers, and non-drummer musicians to judge a series of solo drumming improvisation clips for their level of expressiveness, their positivity (or negativity), and their portrayed emotion among seven categories (happy, sadness, anger, fear, disgust, surprise, and neutral). The clips differed in musical genre, tempo, complexity, drummer’s expressiveness, drummer’s style, and sensory modality (audio-only, video-only, and audio-video). Overall, our results showed that individual differences in musical practise and the high level of familiarity with the music instrument influence how expressiveness and emotions from rhythmic music are perceived, with few exceptions. Regardless of the type and level of musical practise, all groups were able to perceive emotion from music in line with other studies (e.g., Fredrickson, 2000; Vines et al., 2006). For example, all participants could recognise the level of expressiveness communicated by the drummer. That is, all participants, regardless of the level and type of music training, gave higher ratings of expressiveness and perceived happiness, when the performer played with maximum expressive interpretation of the music. In other words, musicianship rarely affected the perceived expressiveness, valence and emotion overall while it did often affect these judgements when specific features of the music were manipulated (e.g., music genre and modality). This lack of a main effect of musical practise is not surprising if one considers that, regardless of the type and level of musical expertise, everyone can perceive emotions from music. However, when specific musical features are manipulated, individual competences can drive the way emotions from music are perceived. That is, non-drummer musicians, drummers, and non-musicians were affected differently by changes in musical genre, tempo, and sensory modality when perceiving expressiveness and a specific emotion from drumming improvisation. For example, all musicians gave a greater weight than non-musicians to the visual information when perceiving the drummer’s expressed emotions. Finally, the effect of musicianship did change with the level of musical experience in that the perceived expressiveness and happiness decreased with increasing years of music training, specifically in the case of jazz and video-only clips.

Non-drummer musicians assigned higher ratings of expressiveness to solo drumming improvisations than non-musicians. Listening to and/or watching a solo drumming performance may be an uncommon experience especially for non-musicians. By contrast, it is likely that these performances are more familiar to non-drummer musicians, such as guitarists or bassists that are used to play together with the drummer. Playing drums or any other musical instrument did not make a difference in the judgement of expressiveness as no difference was found between non-drummer musicians and drummers. Neither did drummers differ from non-musicians, possibly because drummers, due to their background, focused mostly on the technical performance rather than its level of expressiveness.

The drummer’s performances had a similar emotional valence for non-musicians, non-drummer musicians, and drummers. All groups gave more positive than negative responses. Drummers perceived happiness more often than non-drummer musicians, which likely depends on their level of the familiarity with the played instrument. Non-drummer musicians and non-musicians perceived the same level of happiness from music performances supporting previous findings that non-musicians can readily recognise happiness from drumming improvisation (Petrini et al., 2010b) even when the drummer is not instructed to play with a specific emotion in mind. These results suggest that when listening to and/or watching musical performances from our own motor repertoire (e.g., drummers that listen to and/or observe drum performances) more positive emotion are perceived. Indeed, according to the Shared Affective Motion Experience (SAME) model, drummers watching and/or listening to drumming performance are able to access specific information at all levels of the motor hierarchy (the intention level, the goal level, the kinematic level, and the muscle level) and thus imagine emotional intention (Molnar-Szakacs and Overy, 2006; Overy and Molnar-Szakacs, 2009; Molnar-Szakacs et al., 2012). Moreover, studies comparing dancers with specific motor experience, but similar visual experience, with dance actions (Calvo-Merino et al., 2005, 2006) have shown that action representation or areas of the mirror neuron system have a purely motor response. Our results are consistent with this differentiation between visual and motor experience with the portrayed actions and suggest that purely motor experience with the instrument played can enhance the level of emotion perceived and likely cause a specific response of the limbic system (Koelsch et al., 2006; Koelsch, 2009; Petrini et al., 2011).

Despite many studies (e.g., Laukka and Gabrielsson, 2000; Juslin and Laukka, 2003; Juslin et al., 2010) suggesting that music can communicate a wide range of emotions, it was found in our research all participants, regardless of the level and the type of the music training, claimed they perceive mostly neutral and happy emotions from drumming solo improvisation. This finding replicates previous results (Petrini et al., 2010b) on drumming solo improvisation in showing that neutral and happy emotions were amongst the most perceived emotions. However, we did not find the same results for anger, which was another emotion previously shown to be recognised with high accuracy (Petrini et al., 2010b), because of the different music styles used in the present study (i.e., anger was perceived extremely rarely in jazz clips). In fact, in the present study anger was perceived in 15.80% of cases (above chance) in the heavy metal clips as compared to only 3.21% in the jazz clips, thus corroborating the results of Petrini et al. (2010b) when a heavy metal drumming style was used. Given that the musician in our study was not instructed to express specific emotions, participants may have mainly perceived happiness simply because the performer may have chosen to express that particular emotion, rather than any of the others (e.g., sad, anger, fear, disgust, or surprise). The reports of perceiving a neutral emotion could signify that rhythmic music does not convey any emotion in the majority of cases. However, based on the low percentage with which the neutral emotion was chosen (∼37%) and the high level of expressiveness and positivity perceived from the drumming clips this seems improbable. It is more likely that participants could not readily categorise the perceived emotions among the emotion categories given, with the exception of happiness. The possibility that participants could not categorise the emotions based on the choices given is supported by the reports of neutral emotion being largely given when the drummer played with minimal expressive interpretation of the music, suggesting that participants were able to differentiate between high and low emotional performances and perceive changes in expressive intentions (Davidson, 1993).

Drummers, non-drummer musicians, and non-musicians gave different ratings of expressiveness to performances dependent on musical genre. For non-drummer musicians heavy metal and jazz performances had a similar level of expressiveness, while for drummers and non-musicians heavy metal were more expressive than jazz. Moreover, drummers perceived more positive emotions in response to heavy metal than jazz performances. Non-musicians, however, also perceived happiness from heavy metal clips more often than in response to jazz performances, even if it is usually difficult to differentiate happiness from anger (Dahl and Friberg, 2007) as they have similar acoustic features (e.g., fast tempo, high pitch; Juslin, 2009). The finding that non-musicians perceived positive emotions from heavy metal is intriguing and unexpected. Indeed, previous research (Took and Weiss, 1994; Selfhout et al., 2008) often showed that heavy metal music conveys negative thoughts and feelings (e.g., aggression and hostility), and relates to low school performances, antisocial behaviour, drug use, delinquency, and suicidal acts, although this often depends on song lyrics (Anderson et al., 2003) which were not part of this study. Our results are consistent with a recent study (Sharman and Dingle, 2015) which showed that listening to heavy metal music helps fans of this genre to feel positive emotions, enhance their happiness and their well-being in general. Therefore, our findings show that heavy metal music can indeed convey positive emotions to listeners.

Although all participants, in line with previous studies where melodic music was used (e.g., Juslin, 2009), agreed that faster performances were more expressive and conveyed more positive emotions than slower performances, drummers gave higher ratings of expressiveness and perceived positive emotions more often than non-drummer musicians and non-musicians. Our results support those of Wapnick et al. (2004) who found that piano majors were more affected by changes in tempo than non-majors when they judged the tone quality and note accuracy of performances from an international piano competition. Our findings suggest that listeners who share the same type of music training experience with the performer might be more receptive to changes in emotional and sensory information than listeners with different type of musical experience.

Unsurprisingly drummers judged complex rhythms as more expressive than non-musicians. Given their long-term music training with the portrayed instrument drummers are more able to recognise technical aspects of the performance (e.g., odd times, use of the double pedal). In contrast, for non-musicians, simpler music extracts (e.g., 4/4 time) could result in greater pleasure as suggested by the inverted U-hypothesis of music for which overly simple or complex music is less preferred by non-musician listeners (De Meijer, 1989, 1991; Orr and Ohlsson, 2001).

Non-musicians perceived happiness more often for open than crossed arms styles, when compared with drummers. It is indeed well known that arms close to the body communicate negative emotion whereas raised arms communicate joy (De Meijer, 1989, 1991; Dahl and Friberg, 2007). In contrast, drummers perceived more happiness from crossed arms performance than open arms. As discussed before, sharing instrument-specific motor repertoire with the represented music performance may change how the listeners perceive emotion from specific actions (Broughton and Davidson, 2014).

We found that video-only performances were judged as less expressive and conveyed less positive emotion for non-musicians than both groups of musicians. Our results show that the sound in the musical performances was weighted more heavily by all participants than the musician’s body and facial movements when judging the level of expressiveness and positive emotion (Petrini et al., 2010b). However, our results also show that the weight of the visual information (musician’s body and facial movements) increase with music training (Vines et al., 2006). Both non-drummer musicians and drummers gave greater weighting to the visual information than non-musicians and this is likely a consequence of their similar level of visual experience with the drumming performance (Calvo-Merino et al., 2005, 2006). This suggests that both specific motor and visual experience with the instrument enhances the ability to use sensory information when perceiving emotions from music (e.g., Petrini et al., 2009a,b, 2011; Lee and Noppeney, 2011).

The greater the experience of the listeners/viewers (e.g., drummers and non-drummer musicians with many years of music training) the less they perceived expressiveness from jazz, video-only clips and happiness from jazz music, most likely because they focused on the technical features of these drumming performances. However, in previous studies (e.g., Bhatara et al., 2011; Lima and Castro, 2011a; Castro and Lima, 2014) where melodic music was used it was found that years of music training were associated with enhanced recognition of musical expressiveness and emotions from audio-only music excerpts. These contrasting results might depend on the use of rhythmic, rather than melodic, music in the current study and on the manipulations of additional music features such as musical genres and sensory modalities, which were not previously investigated (e.g., Bhatara et al., 2011; Lima and Castro, 2011a; Castro and Lima, 2014). Indeed, the results between our study and the mentioned research align for the heavy metal style of music and when audio-only or audio-video performances were used, both finding no significant decrease in perceived expressiveness and emotion with increasing years of musical experience.

Our results can contribute to the suggested theoretical framework named BRECVEMA (Juslin, 2013). This model unifies a set of eight mechanisms of emotion induction (e.g., Brain stem reflex, Rhythmic entrainment, Evaluative conditioning), that mediate between different musical features and the resulting induced emotions in listeners/viewers. This mediation mechanism can explain both why a given event arouses an emotion and why the aroused emotion is of a certain kind. For example, the Rhythmic entrainment mechanism explains that a piece of music evokes an emotion because a powerful rhythm in music influences some internal bodily rhythm in the listener (e.g., heart rate). Each of these mechanisms can induce emotion from music and in many instances more than one mechanism is involved when emotion is derived from music (Juslin et al., 2013). Our results examined emotional perception and thus we cannot definitely conclude that long-term music training or expertise would affect the felt emotions, however, our results do suggest that long-term practise or expertise may be an additional emotional inducement mechanism, since musicians did perceive higher levels of emotion and expressiveness than non-musicians in many instances. Future studies could examine this possibility by using both melodic and rhythmic music and testing emotion inducement in individuals with different levels and types of musical training. For example, reports of felt emotions as well as physiological responses could be used in future studies to examine whether the perceived expressiveness and/or emotion is also felt (Juslin and Laukka, 2004).

Finally, our findings have potential implications for music therapy and clinical practise. They suggest that drumming could be used to enhance the perception of happiness in young children and infants that find judging emotion difficult based on other non-verbal cues (e.g., facial expression, voice prosody, and body movements). This is based on the finding that drums are an approachable instrument that have been shown to affect cognitive abilities from an early age (Gerson et al., 2015). Also, since practising a music instrument has been shown to produce more “joy,” “emotional synchronicity,” and “initiation of engagement” in autistic children (Kim et al., 2009), a period of training with drums could also have positive effects on these individuals’ emotional state and understanding. Finally, since our results show that long-term music training enhances the ability to perceive emotions from facial and body information during the musician’s performance, training with a music instrument (not specifically the drums) would be beneficial to individuals that have difficulties in using this type of information when judging emotions in others.

### Limitations

Our study is a quasi-experimental because random assignment of the participants to the three groups (non-musicians, drummers, and non-drummer musicians) was not possible, since each group consisted of people with specific individual differences. However, in future researches, non-musicians, after a period of music training, could be randomly assigned to various groups in order to support our results by using an experimental method. Moreover, we did not match the groups for other relevant variables (e.g., education, working memory capacity, and auditory abilities). Since it is well established that cognitive (e.g., working memory) or auditory (e.g., pitch and rhythm perception) abilities can be affected by musical training (e.g., Moreno et al., 2011; Slevc et al., 2016), future studies should better match participants for possible confound variables.

In addition, there are other limitations to this study that should be acknowledged. Firstly, we examined only a rhythmic instrument with the decision to study drums based on evidence that the upper-body movements permitted by drumming are more evident and less expressively restricted than those permitted by other instruments (Petrini et al., 2010b). Additionally, playing drums could be a very effective means of therapy (Overy, 2003; Zentner and Eerola, 2010; Wan et al., 2011; Gerson et al., 2015). However, it is unknown whether our results are transferrable to other percussion and melodic musical instruments, for example an instrument like piano (Lee and Noppeney, 2011) that allows a good range and variety of upper-body movement while maintaining the melodic component of the music would be essential in future studies to understand the effects of long-term music training on perceived emotion from melodic and rhythmic music. However, this limitation is also one of the novelties of the present study as knowing that training with a purely rhythmic instrument can affect how we perceive emotion from music is important and encouraging in terms of clinical applications using rhythmic music instruments.

We chose to use a single drummer as performer as we had many factors to examine and having one musician limited the duration of the experiment, which would otherwise have been unsustainable, especially when examining perceived emotion. However, this is a main limitation of our study, as other performers may have chosen to improvise in different ways, thereby leading to different expressive and emotional content. Besides, as mentioned above, no instructions concerning specific emotional intentions were given to the drummer. We cannot rule out that these choices may have influenced the results. Indeed, only two emotion categories (neutral and happy) were selected above chance levels. It seems difficult to discern whether or not neutral and happy rates reflect a bias toward using these emotion categories more often than others (Isaacowitz et al., 2007). Future studies could focus on the musical features where differences were found between musicians and non-musicians (e.g., musical genre, tempo, sensory modality) and include a higher number of performers.

Finally, the forced-choice emotion perception task (e.g., asking participants to choose the perceived emotion by selecting only one from seven basic emotion categories) is a limitation because it can have an impact on statistical analyses, such as increased co-linearity (if one response is chosen, the others are not) generating high perception rates artificially (Cornwell and Dunlap, 1994; Frank and Stennett, 2001). Future studies could include more complex emotions such as pride, jealousy and contentment, other scales such as the Self-Assessment Manikin (SAM; which makes use of a non-verbal pictorial assessment technique rather than emotional labels), and/or allow participants to choose more than one emotional category.

## Conclusion

The presented research contributes to the understanding of the effect of long-term musical training on emotional perception by showing that individual differences in musical training (e.g., motor and/or visual experience with a specific instrument) influence the way expressiveness and emotions from solo drumming improvisations are perceived. Non-musicians, non-drummer musicians, and drummers were affected differently by changes in some characteristics of the music performance such as musical genre, tempo, and modality, when perceiving expressiveness, valence, and emotion from drumming improvisation. Therefore, long-term musical training shapes several cognitive and perceptual abilities (e.g., Petrini et al., 2009a,b, 2010a; Lee and Noppeney, 2011; Lee and Noppeney, 2014), and also emotional processing from purely rhythmic music. This has potential implications for music therapy, clinical practise, and theory of emotion inducement from music.

## Author Contributions

MDM, MG, and KP designed the experiments, wrote and reviewed the manuscript. ET performed the statistical analysis.

## Conflict of Interest Statement

The authors declare that the research was conducted in the absence of any commercial or financial relationships that could be construed as a potential conflict of interest.

## Supplementary Material

VIDEO 1 Example of audiovisual clip used in the present study: heavy metal, simple rhythm, slow tempo, maximum expressive interpretation, crossed arms.

VIDEO 2 Example of audiovisual clip used in the present study: jazz, complex rhythm, fast tempo, minimal expressive interpretation, open arms.
